# Modulation of lipid metabolism by exercise: exploring its potential as a therapeutic target in cancer endocrinology

**DOI:** 10.3389/fendo.2025.1580559

**Published:** 2025-05-23

**Authors:** Haodong Liu, Tong Yang, Seongbeom Choi

**Affiliations:** College of Arts & Physical Education, Gangneung-Wonju National University, Gangneung-si, Gangwon-do, Republic of Korea

**Keywords:** cancer, lipid metabolism, exercise, lipid oxidation, cancer treatment

## Abstract

Cancer progression is tightly linked to metabolic changes, particularly in lipid metabolism, which is crucial for tumor growth and metastasis. Exercise, known for its health benefits, is gaining recognition for its ability to influence cancer-related lipid metabolism. Metabolic shift prioritizes lipid oxidation over glucose metabolism, hence limiting the energy supply available to tumor cells and reducing their metabolic adaptability. Exercise also enhances mitochondrial function and aids the immune system, further bolstering its anti-cancer effects. Additionally, exercise mitigates cancer-related symptoms like fatigue, improves insulin sensitivity, and counteracts metabolic issues such as cachexia. Despite promising insights from studies, challenges persist in comprehending the molecular mechanisms of exercise’s impact on lipid metabolism in cancer. Future research should aim to identify optimal exercise regimens for cancer patients, explore the combined effects of exercise and cancer treatments, and delve into the molecular pathways connecting exercise with tumor suppression. With its potential benefits, exercise could act as a supportive therapy alongside conventional cancer treatments, enhancing patient outcomes and quality of life.

## Introduction

**Figure 1:**
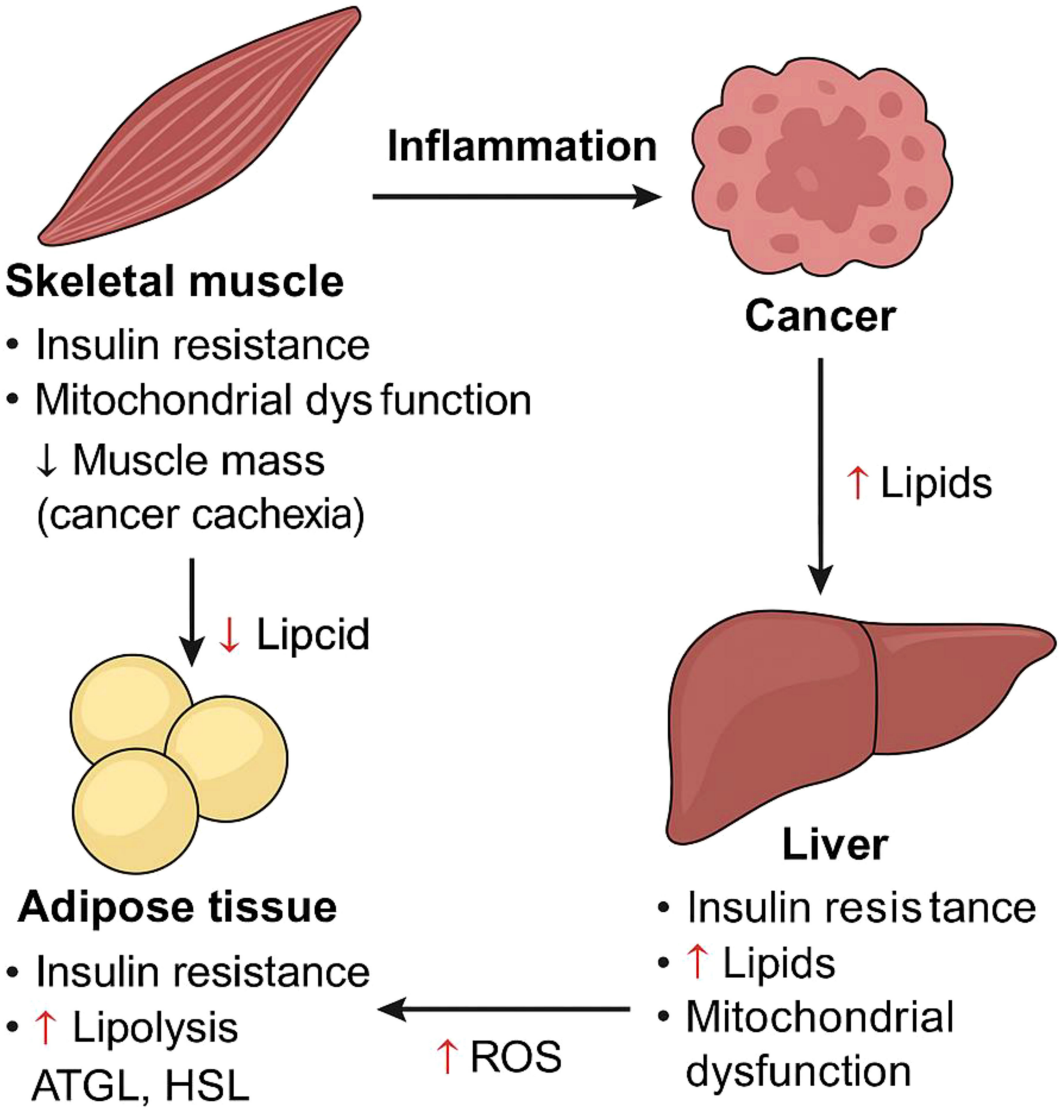
Diagram illustrating how cancer induces metabolic dysfunction across key tissues, including skeletal muscle (myocytes), adipose tissue (adipocytes), and the liver (hepatocytes). Cancer-driven systemic changes lead to insulin resistance, elevated circulating lipid levels, mitochondrial dysfunction, chronic inflammation, and progressive muscle wasting—collectively contributing to cancer cachexia. The metabolic crosstalk between these tissues exacerbates disease progression. Key molecular players include adipose triglyceride lipase (ATGL), hormone-sensitive lipase (HSL), and reactive oxygen species (ROS).

Cancer stands out as one of the most deadly illnesses, exhibiting particularly high incidence and mortality rates when compared to other non-communicable diseases (1). In recent decades, there have been significant advancements in cancer treatment. Nonetheless, the majority of cancer patients, particularly those in advanced stages or with metastases, still tend to have a limited lifespan. Understanding the molecular processes that drive tumor growth and devising more effective clinical approaches for treating cancer are crucial (2). Cancer influences the body by disrupting various pathways, such as inflammation, mitochondrial dysfunction, cachexia, and other metabolic-related alterations (**Figure 1**).

In 2016, the percentage of adults who were physically inactive in wealthier countries (36.8%) was more than double the percentage observed in poorer countries (16.2%). In many nations, particularly in areas such as the Eastern Mediterranean and the United States, women generally engage in fewer physical activities compared to men (3). On the other hand, the onset of cancer has been associated with a mix of genetic components and environmental factors. Studies carried out both in laboratories and through observational methods suggest that certain lifestyle choices, such as not getting enough exercise, smoking, dietary habits, and alcohol consumption, may influence the chances of cancer recurrence and survival after a cancer diagnosis (4).

Research suggests that engaging in regular physical activity may reduce the likelihood of developing various forms of cancer (5). This encompasses breast cancer in women who have gone through menopause, colon cancer, and possibly cancers of the prostate, uterine lining, lungs, and pancreas (6). Regular exercise has been associated with up to a 40% decrease in the incidence of these types of cancer (7). Engaging in physical activity appears to significantly enhance bodily strength and muscle function, while also positively impacting various facets of a person’s mental well-being. Participating in physical exercise can impede tumor development through various processes, including (a) improving blood vessel development and circulation, (b) strengthening the immune system’s function, (c) altering the metabolic activity of tumors, and (d) influencing the relationship between muscles and cancer (8). Preliminary indications of these mechanisms have appeared, yet additional intervention studies are necessary to confirm a causal connection between these mechanisms and the regulation of tumor growth and progression (9). A less frequently explored area of research in exercise science is the changes in how lipids are metabolized. Physical activity has the potential to greatly alter lipid metabolism in individuals with cancer. It affects how cancerous cells obtain and use lipids, which may help slow down tumor development and boost treatment effectiveness. This primarily occurs by boosting fatty acid oxidation in muscle tissue, which reduces lipid levels in the bloodstream accessible to cancer cells, while also strengthening the immune reaction in the tumor’s nearby environment (10). Investigating how lipid metabolism influences cancer progression and the role of exercise as a regulatory factor offers a robust foundation for a review article. Lipid metabolism is crucial in cancer biology because tumor cells often rely on altered pathways to support their rapid expansion and dissemination (11). These variations are essential for energy production, the formation of cell membranes, and the impact on the tumor microenvironment, highlighting the importance of research in this area (12). Engaging in physical activity significantly impacts lipid metabolism by increasing fat breakdown, improving insulin sensitivity, and altering the lipid profile. Consequently, this boosts HDL (beneficial cholesterol) levels and lowers LDL (harmful cholesterol) and triglyceride levels (13). These findings indicate that consistent exercise might alter lipid metabolism in a manner that could slow down cancer progression, possibly serving as an auxiliary therapeutic approach in conjunction with conventional treatments (14). A thorough analysis would integrate current studies on how exercise affects lipid metabolism in cancer, pinpoint areas lacking information, and propose directions for future investigations. It would also explore the potential of developing targeted exercise programs to improve therapeutic treatments, offering a holistic approach to cancer management. This article aims to provide an overview of how physical activity influences lipid metabolism in cancer, propose mechanisms that may explain its therapeutic advantages, and examine the potential benefits of integrating exercise into cancer treatment strategies.

## Lipid metabolism in homeostasis

Lipids, recognized for their ability to repel water, encompass glycolipids, sterols, phospholipids, monoglycerides, diacylglycerides, and triglycerides (15). A wide variety of lipids originate from fatty acids, which are diverse compounds made up of extended hydrocarbon chains. These chains differ in the count of carbon atoms and their saturation level or the presence of double bonds. Mammals have the ability to synthesize specific fatty acids, especially those that feature double bonds at or before the delta-9 position along the hydrocarbon chain. Some fatty acids, especially polyunsaturated ones (PUFAs), are vital and must be obtained from the diet (16). Helpful gut bacteria mainly produce short-chain fatty acids, which are distinguished by having fewer than six carbon atoms, unlike medium- and long-chain fatty acids (17). Fatty acids play a vital role as essential components of membrane lipids. These molecules, possessing both water-attracting and water-repelling characteristics, serve as the essential building blocks of biological membranes. Glycolipids play a role in recognizing cells, controlling inflammation, and interacting with signals from the immune system. Cholesterol is an important membrane lipid characterized by its structure of four interconnected hydrocarbon rings (18). It is essential for managing membrane fluidity and creating microdomains, and it also acts as a precursor in steroid hormone synthesis (19, 20). In addition to their roles in energy storage and membrane formation, fatty acids act as precursors for lipid mediators that function as signaling molecules (21). Arachidonic acid, a variety of polyunsaturated fatty acid derived from omega-6, serves as the initial substance for the production of eicosanoids (22). This includes the synthesis of prostaglandins and thromboxanes through the cyclooxygenase (COX) pathway, along with the formation of leukotrienes via the lipoxygenase route. Prostaglandins, including prostaglandin E2 (PGE2), play a role in triggering inflammation in tissues and creating an environment that encourages tumor development (23).

**Figure 2:**
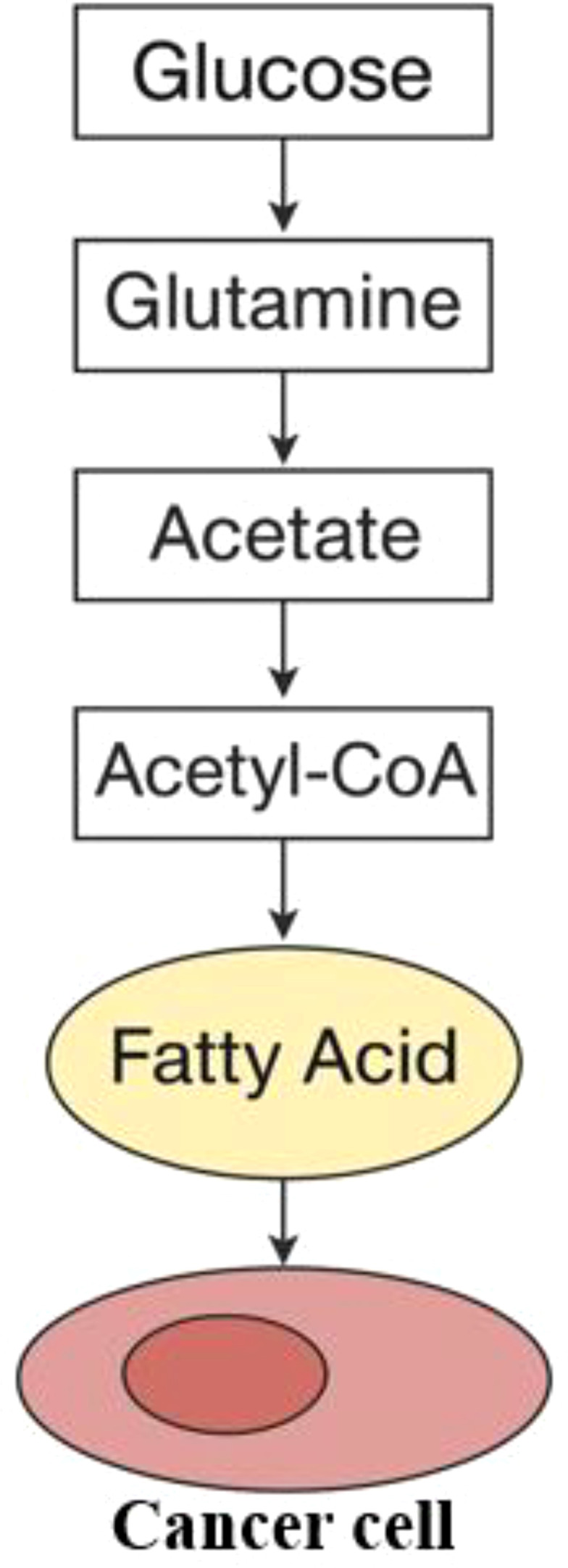
Interaction of lipids in cancer cells. The formation of fatty acids starts with acetyl-CoA, which can be derived from glucose, glutamine, or acetate.

Eicosapentaenoic acid (EPA) and docosahexaenoic acid (DHA) are forms of omega-3 fatty acids, classified as polyunsaturated fatty acids (PUFAs), essential for efficient cellular communication. These acids generally aid in reducing inflammation and are believed to lower the risk of breast cancer as well as several other types of cancer (24). Lipid mediators originate from essential fatty acids, and their availability is significantly influenced by dietary intake. Moreover, phospholipases can release them from the lipids present in membranes (25). Different kinds of phospholipase A (PLA) enzymes release free fatty acids from phospholipids by cleaving the ester bond at the sn-1 or sn-2 position. The distinct choices for saturated versus unsaturated fatty acids in these areas lead to unique PLA isoforms that specifically affect how various free fatty acids are distributed (25). Lysophospholipase D can modify residual lysophospholipids to produce lysophosphatidic acid (LPA), a molecule that participates in various signaling activities (26). Lysophosphatidic acid (LPA) engages with at least six unique G protein-coupled receptors (GPCRs), triggering the RAS, PI3K, RAC, and RHO signaling pathways, which promote cellular mobility and survival. Each receptor shows a specific affinity for LPA molecules that have varying lengths of acyl chains and different saturation levels (27, 28) (**Figure 2**).

## Alteration of lipid metabolism in cancer

While most somatic cells obtain lipids from the diet or are synthesized by the liver, some cancer types initiate the *de novo* lipogenesis pathway, which decreases their dependence on external lipid sources (29). Within the cytoplasm, acetyl-CoA, which is obtained from sources such as glucose, glutamine, or acetate, acts as the essential precursor for the creation of fatty acids (30). The enzymes known as acetyl-CoA carboxylase, also called ACC1 and ACC2 or ACACA and ACACB, are essential for transforming acetyl-CoA into malonyl-CoA. Following this, fatty acid synthase (FASN) promotes a series of condensation reactions, which eventually result in the creation of palmitate, a saturated fatty acid made up of a 16-carbon chain. Palmitate undergoes elongation facilitated by fatty acid elongases (ELOVL1-7) and is subsequently desaturated by stearoyl-CoA desaturases (such as SCD and SCD5 in humans) or by fatty acid desaturases (FADS1-3). This series of transformations creates the cellular reservoir of non-essential fatty acids, such as oleate (C18:1), an 18-carbon monounsaturated fatty acid. The link between heightened fatty acid synthesis and various cancers is well established, with numerous studies indicating that the process of lipid production is crucial for the development of tumors (31). Certainly, here’s a paraphrased version: Diverse signaling pathways that cause cancer eventually result in fatty acid production. The PI3K/Akt signaling pathway boosts the synthesis of enzymes required for fatty acid production and promotes the phosphorylation and activation of ATP-citrate lyase (ACLY), which is the enzyme that converts cytoplasmic citrate into acetyl-CoA (32, 33). Conversely, the creation of fatty acids is suppressed by AMP-activated protein kinase (AMPK). Inhibition is controlled by the phosphorylation of ACC, a process influenced by the STK11/LKB1 tumor suppressor pathway (34). Although initial research showed that cancer cells synthesize fatty acids from scratch, they also determined that these cells need to acquire at least some lipids from their surrounding environment (35). Lipids can be absorbed through multiple mechanisms, including the uptake of low-density lipoprotein (LDL) particles via the LDL receptor (LDLR). The groundbreaking research conducted by Goldstein and Brown first described this process (36). Additionally, cells absorb free fatty acids (FFAs) through two mechanisms: either via the CD36 fatty acid translocase or through fatty acid transport proteins that belong to the solute carrier family SLC27 (37). Fatty acid-binding proteins (FABPs) aid in the assimilation of fatty acids by supporting their intake and transportation (38). The increase in production of new fatty acids within cancer cells alters the composition of cell lipids, presenting opportunities for diagnostic use (39). In addition, it reduces the uptake of polyunsaturated fatty acids (PUFAs) and simultaneously increases the concentration of saturated fats and monounsaturated fatty acids (MUFAs) within the membrane lipids. This protects against harm caused by lipid peroxidation, a process in which polyunsaturated fatty acids (PUFAs) undergo oxidation due to the action of reactive oxygen species (ROS) (40). The study discovered that using Soraphen A to block fatty acid production altered cell membrane characteristics, increasing the susceptibility of cancer cells to oxidative stress-induced death (40). The uptake of fatty acids is critical in the advancement of cancer, with external factors like palmitic acid shown to increase cell movement and promote the dispersal of secondary tumors in squamous cell carcinoma (41). Likewise, blocking CD36 to prevent the absorption of fatty acids has demonstrated potential therapeutic advantages in early-stage research models of prostate cancer (42). The different types of lipids found in the external extracellular environment also influence the extent to which *de novo* synthesis and uptake contribute. While the dietary fats a person consumes may play a role, it is primarily the differences in the tumor’s surrounding environment—such as limited blood flow—that dictate how accessible lipids are in that region (43) (**Figure 2**).

In cancer cases, lipids play roles in numerous processes that have the potential to be disrupted. Many research studies have investigated the impact of inhibiting lipid production on cancer cell survival and tumor progression (reviewed in (44, 45)). Researchers have developed and tested compounds aimed at FASN in various cancer models (reviewed in (46)). Furthermore, utilizing the allosteric inhibitor ND-646, which specifically targets ACC1 and ACC2, led to a decrease in tumor growth in Kras/p53-/- and Kras/Stk11-/- mouse models of non-small-cell lung cancer. This effect was observed both when the inhibitor was used independently and in conjunction with carboplatin (47). Besides their role in maintaining cell membrane structure, lipids also contribute to cancer-related characteristics that support cellular transformation and tumor progression. For example, sphingolipids are essential for managing cell-to-cell communication and maintaining cell viability (48). In the sections that follow, we will concentrate primarily on how FAs contribute to energy metabolism, respond to stress, and support survival in cancer. We will also explore recent studies linking changes in lipid makeup to ferroptosis, metastasis development, stem cell characteristics, and diverse interactions within the tumor microenvironment.

Energy and fat-related metabolic processes: The resurgence of fatty acid synthesis is gaining recognition as an essential factor in the metabolic changes occurring during cellular transformation. The importance of fatty acid oxidation for the survival of cancer cells in many forms of cancer is becoming more apparent. Numerous cancer types have been found to show elevated levels of FAO enzymes (49) Furthermore, inhibiting FAO results in reduced tumor growth in various cancer models. In studies using orthotopic xenografts from patients with triple-negative breast cancer, blocking carnitine palmitoyltransferase 1 (CPT1), the key enzyme that controls fatty acid oxidation, resulted in reduced tumor growth and prolonged survival periods (50) and a glioblastoma model located in its original anatomical position (51). Enzymes that act before CPT1 have been recognized as crucial for tumor development. An enzyme called ACSL3 (acyl-CoA synthetase long chain 3) is essential for transforming free fatty acids into fatty acyl-CoAs. The molecules produced by this process can subsequently be used as starting materials for lipid synthesis or for breaking down fatty acids. Lung cancers with the KrasG12D mutation exhibit higher levels of ACSL3 expression. Removing this gene greatly reduces fatty acid absorption, alters fatty acid metabolism, and decreases tumor growth (52). In cases of human glioblastoma, there is a significant elevation in acyl-CoA-binding protein (ACBP) levels. This protein engages with medium- and long-chain fatty acyl-CoA molecules and may act as a transporter or provide structural support for these molecules. Lowering its levels impairs fatty acid breakdown, resulting in aging of both orthotopic xenograft and genetically induced glioblastoma mouse models (53). In certain types of cancer, fatty acid oxidation is stimulated by particular oncogenes, like c-Myc in triple-negative breast cancer (50) or Kras mutation in lung cancer (52), to promote growth and development. Additionally, FAO plays a critical role in supplying NADPH (54–56), Especially during times of increased energy requirements, such as when glucose is limited or growth occurs without attachment, the conditions can hinder the generation of NADPH through the pentose phosphate pathway (54). Mechanistically, this process depends on AMPK, which inactivates ACC by attaching a phosphate group. This action hinders the synthesis of fatty acids, a process that heavily utilizes cytosolic NADPH. At the same time, it promotes fatty acid breakdown since malonyl-CoA, the substance generated by ACC, serves as an allosteric inhibitor of CPT1. The specific mechanism through which acetyl-CoA, produced from the breakdown of fatty acids, contributes to the creation of NADPH in the cytosol remains not entirely clear. The molecule is transported into the cytosol in the form of citrate and subsequently converted into α-ketoglutarate by the enzyme isocitrate dehydrogenase 1 (IDH1) (57).

Oleic acid (C18:1), a typical monounsaturated fatty acid present in cells, is produced when the enzyme stearoyl-CoA desaturase (SCD) aids in converting stearic acid (C18:0) through a desaturation reaction. This process is essential for preserving the right balance of saturated and unsaturated fatty acids in cells. When there is a shortage of lipids, blocking SCD leads to stress in the endoplasmic reticulum (ER) and triggers cell death. However, the incorporation of oleic acid or other unsaturated fatty acids can reduce these effects (58–61). Moreover, targeting SCD has proven to successfully reduce tumor development in various cancer models (62–65). While essential for cancer cell survival, particularly in lipid-deficient environments, blocking desaturation interferes with mitochondrial function and heightens oxidative stress. This implies that ER stress might be a secondary consequence of compromised mitochondrial respiration (59, 60). This resource highlights the intricate role of lipid metabolism in cancer and its potential as a therapeutic target. Cancer cells’ reliance on external lipid sources can change depending on nutrient and oxygen availability, as well as the specific oncogenic signaling pathways active within the tumor. Cells affected by oncogenes such as AKT/mTOR or Ras exhibit differing reliance on lipid uptake and lipid biosynthesis (66, 67). For example, cells with H-RasV12G or K-RasG12D mutations tend to rely on taking in lipids, while cells with activated AKT pathways are more reliant on creating lipids from scratch.

A lipid modification process guarantees a suitable equilibrium between saturated and unsaturated fatty acids within membrane phospholipids. This involves the removal and addition of acyl groups via the Lands’ cycle, with the help of enzymes such as lysophospholipid acyltransferases (LPLATs) (68). Overhauling this process is essential for enhancing cellular responses to stress and altering the signaling pathways that govern cell survival and programmed cell death. In ferroptosis research, a type of cell death triggered by the buildup of lipid peroxides, the makeup of membrane lipids plays a crucial role. Enzymes such as ACSL4 and LPCAT3, responsible for incorporating polyunsaturated fatty acids into membrane lipids, are essential in determining susceptibility to ferroptosis (69, 70). These discoveries underscore the possibility of altering lipid metabolism pathways to trigger ferroptosis, which could enhance the destruction of cancer cells. Moreover, lipids play an essential role in cancer progression because changes in lipid metabolism are closely associated with the capacity of cancer cells to move, infiltrate tissues, and establish metastases. Enzymes such as MAGL and SOAT1, involved in lipid metabolism, are observed to be significantly expressed in more aggressive forms of cancer. This increase supports metastasis by improving lipid processing and the associated signaling pathways (71, 72). Elevated levels of lipid transporters like CD36 are associated with a greater capacity for metastasis, emphasizing the crucial role of lipid absorption in supporting the spread of cancer cells (41, 73).

The connection between lipid metabolism and cancer progression is intricate, involving a variety of enzymes and pathways that facilitate cell survival, growth, and the spread of cancer within the body. These pathways offer numerous targets for therapy, potentially resulting in the development of strategies that specifically impede the proliferation and survival of cancer cells while sparing normal cells. Recent studies are uncovering the complex connection between fat metabolism and the biology of cancer, emphasizing the crucial influence that lipids have on the outcomes of cancer cells. This suggests that lipids serve functions beyond merely acting as structural components or energy stores (43).

## Lipid metabolism during exercise

When the body is at rest, more fatty acids are usually released from fat reserves than are being metabolized. The speed at which fatty acids are released into the bloodstream is typically twice as fast as the rate at which they are broken down (74). Consequently, a large portion of fatty acids, which originate from the degradation of fat tissue triacylglycerols, is largely reconverted into triacylglycerols, predominantly by the liver (75). Participating in low to moderate intensity physical activities, using 25-65% of your maximum oxygen intake (V̇O2max), can boost your body’s rate of burning fat by 5 to 10 times compared to resting levels (76) due to the heightened energy requirements of muscles and the greater availability of fatty acids. A significant part of the enhanced fatty acid supply comes from the breakdown of adipose tissue triacylglycerols through lipolysis, which increases by two to three times (75). High-density lipoprotein is essential for lipid transport within the blood. It helps decompose chylomicrons and very-low-density lipoproteins (VLDL) and plays a role in removing excess free cholesterol from these lipoproteins. The quantity of chylomicron particles does not alter after engaging in either short-term or long-term aerobic activities (77, 78). Unexpectedly, following a six-month resistance training regimen, adults with diabetes experienced a notable decrease in apo B48 levels, a substance found in chylomicrons and their remnants (79). Additionally, strength training lowers the triglyceride and cholesterol content in chylomicrons in healthy, sedentary men (80), along with the integration of fatty acids produced within the body and those obtained from the diet into chylomicron triglycerides and lipoproteins rich in triglycerides in overweight or obese men who have prediabetes (81). Participating in a combination of cardio workouts and strength training can lower total cholesterol and LDL-C levels and boost high-density lipoprotein (HDL-C) cholesterol levels (82, 83).

Highlighting its essential function in managing fat and energy equilibrium, lipolysis occurs in nearly every kind of tissue and cell (84). When working out at 45% to 65% of VO2max, fatty acids serve as the primary energy source. These fatty acids come from stored body fat, lipids present in muscle tissues, and the food you eat (85). When engaging in activities of low to moderate intensity or during prolonged periods of exercise, skeletal muscles mainly utilize fatty acids as their primary energy source, with glucose oxidation playing a smaller role. In contrast, glucose is the primary energy source during brief periods of intense physical activity (86). Therefore, as the intensity of physical activity rises, the reliance on fat within the overall oxidative metabolism diminishes (87, 88). Maximal oxygen consumption, often referred to as maximal aerobic capacity or maximal oxygen uptake (VO2max), serves as the metric for evaluating training load. This represents the highest quantity of oxygen an individual can utilize within a given time frame during exercise. VO2max differs greatly from person to person, influenced by factors such as aerobic fitness level, genetics, age, health, and gender. It describes the functional aerobic capability of one person during a particular exercise activity and indicates their level of cardiorespiratory fitness (89). As the intensity of exercise rises, the body shifts from oxidizing fatty acids to glucose, resulting in a decrease in fat oxidation’s contribution to total energy and an increase in the use of carbohydrates. When exercise intensity exceeds roughly 80% of VO2 max, carbohydrates become the main energy source (90–92). When exercising at 65% of VO2max, the body reduces its dependence on plasma fatty acids and instead increases its use of intramuscular triglycerides (IMTG). This shift allows IMTG to supply roughly half of the fatty acids needed for complete fat oxidation (93, 94). The breakdown of fats in peripheral regions and the rate at which free fatty acids enter the bloodstream are most accelerated during activities involving minimal physical exertion. As exercise becomes more intense, the stimulation gradually reduces until reaching a threshold. When you reach 85% of your VO2max, there is a notable decrease in the levels of free fatty acids in your bloodstream.

**Figure 3:**
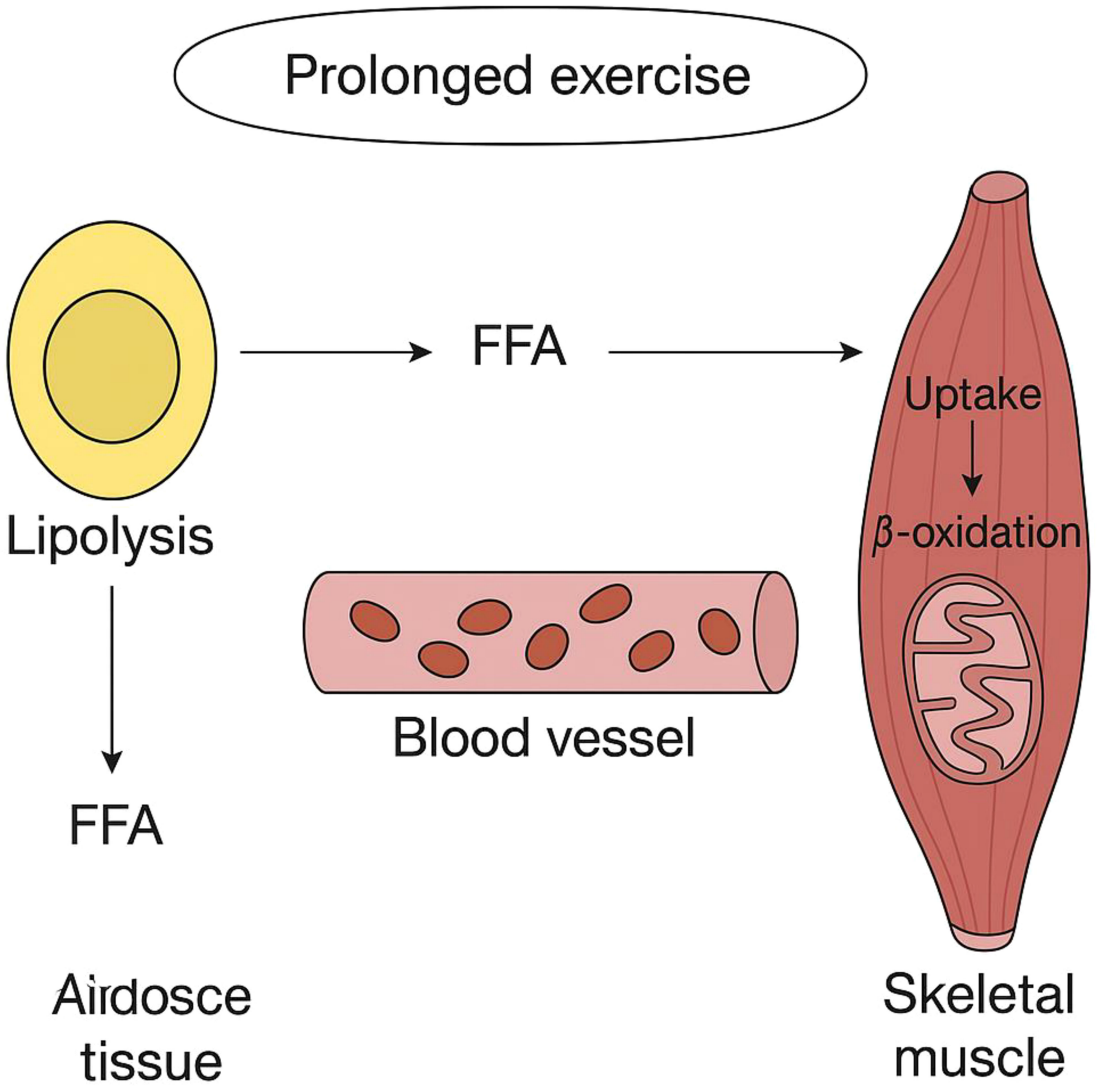
Depicts how fatty acids are released and utilized in skeletal muscles during extended periods of exercise.

Once exercise intensity surpasses roughly 50% of maximum oxygen uptake (VO2max), the body begins to depend more heavily on muscle glycogen. Additionally, at higher exercise intensity levels, carbohydrate oxidation is greater in comparison to moderate-power exercise outputs (95). The precise chain of molecular activities occurring during physical exercise results in the breakdown of fatty acids within the skeletal muscles. Research indicates that the amount of acetyl-CoA present in the mitochondrial matrix influences fatty acid oxidation, and these effects vary depending on how intense and how long the exercise is (96). The speed of glycolysis appears to be key in influencing the amount of acetyl-CoA within the mitochondria, which in turn impacts the control of fatty acid metabolism (97). During strenuous aerobic exercises, glycolysis accelerates, leading to a higher production of pyruvate. As a result, there is an accumulation of acetyl-CoA, which is then moderated by the enzyme catalase (CAT). This alleviates the limitation on the pyruvate dehydrogenase complex (PDH), enhancing the transformation of glucose into energy and facilitating ATP production (98). Transforming fatty acids into fatty acyl-CoA esters is an essential step for their subsequent absorption into the mitochondria. This alteration helps retain fatty acids within the cell and creates a gradient as well. The process of converting fatty acids into acyl-CoA molecules is controlled by the enzyme acyl-CoA synthetase (ACS). Skeletal muscle cells comprise various ACS isoforms, each situated in unique subcellular regions and preferring particular fatty acids. The ACSL1 variant is crucial for the metabolism of fatty acids to generate energy in skeletal muscles during physical activity (86). The majority of free fatty acids that originate from the diet or are released from stored fat consist of long-chain fatty acids. In contrast to short- and medium-chain fatty acids, they cannot directly pass through mitochondrial membranes. Rather, the enzyme referred to as carnitine palmitoyl transferase 1 (CPT1) transforms them into compounds known as fatty acyl carnitines (refer to **Figure 3**). This enzyme, found on the mitochondrial outer membrane, appears in two forms: one connected to the liver (L-CPT1) and the other related to muscle tissue (M-CPT1). The M-CPT1 variant is the predominant form present in skeletal muscle (99). Acyl carnitine moves across the mitochondrial inner membrane via a translocase called CACT, which swaps it with a free carnitine molecule. Once inside the mitochondrion, CPT2 transforms it back into acyl-CoA for oxidation (100) (**Figure 3**). As exercise intensity increases, the concentrations of acetyl-CoA and acetylcarnitine in the muscles can significantly elevate, potentially reaching three times the levels found during rest or mild activity. Meanwhile, the proportion of free carnitine experiences a significant drop, decreasing from constituting 75% of the overall carnitine in muscles while at rest to only 20% during physical activity at 90-100% of maximal oxygen uptake (VO2max). The findings indicate that acetylcarnitine is a significant byproduct produced during intense muscle exertion. Additionally, carnitine contributes to keeping the equilibrium between acetyl-CoA and CoA stable by reducing excess acetyl groups (101, 102) (**Figure 3**).

Epinephrine, norepinephrine, and glucagon promote the breakdown of triglycerides in fat tissue, leading to the release of fatty acids, while insulin opposes these actions (103). When epinephrine and glucagon attach to their respective receptors on the exterior of fat cells, they trigger the activation of adenylyl cyclase (104). Following this, the enzyme produces cyclic AMP (cAMP). Cyclic AMP activates protein kinase A (PKA), leading to the phosphorylation of both hormone-sensitive lipase (HSL) and perilipin located on the lipid droplet surface (105). Increasing the phosphorylation of perilipin boosts ATGL activity, resulting in more diacylglycerol (DAG) being accessible for hormone-sensitive lipase (HSL) to utilize. Hormone-sensitive lipase converts diacylglycerol (DAG) into a free fatty acid (FFA) and monoacylglycerol (MAG) (106). The enzyme monoacylglycerol lipase breaks down monoacylglycerol. In fat cells, fatty acids are carried to the cell membrane by attaching to a particular protein that facilitates their transport. Afterward, they leave the cell and bind to serum albumin found in the bloodstream. (b) Engaging in physical activity promotes the production of lipoprotein lipase (LPL) on the external surface of endothelial cells in skeletal muscles. When lipoprotein lipase (LPL) activity rises, it accelerates the breakdown of triglycerides within triglyceride-rich lipoprotein particles, like very-low-density lipoproteins and chylomicrons (107). This process leads to the liberation of phospholipids, unbound cholesterol, glycerol, and non-esterified fatty acids. Cholesterol that undergoes esterification becomes part of the central region of HDL particles, leading to higher HDL-C levels in the bloodstream. (c) Free fatty acids (FFA), produced from the decomposition of lipoproteins and fat tissue, separate from albumin and are transported into muscle cells through dedicated mechanisms designed for fatty acid transport. This includes fatty acid translocase (FAT/CD36), plasma membrane-associated fatty acid binding proteins (FABPpm), and proteins dedicated to the transport of fatty acids (FATP). Among these are the fatty acid translocase (FAT/CD36), fatty acid binding proteins associated with the plasma membrane (FABPpm), and proteins that play a role in the transportation of fatty acids (FATP) (108). One possible sequence of events involves fatty acids initially attaching to FAT/CD36 before being transferred to fatty acid transport proteins (FATP) or cytosolic fatty acid-binding proteins (FABP). Inside the cell, the enzyme acyl-CoA synthetase (ACS) starts the activation process by transforming them into acyl-Coenzyme A (acyl-CoA) (109). Carnitine palmitoyl transferase 1 (CPT1) assists in transporting acyl-CoA esters into the mitochondria, where they undergo degradation via β-oxidation. The citric acid cycle converts acetyl-Coenzyme A into energy, which is then stored as ATP. This energy reserve fuels muscle contractions and a range of other energy-requiring metabolic activities inside the muscle cell. In this kind of workout, intramuscular triacylglycerol stores release fatty acids with the help of hormone-sensitive lipase in the muscles, aiding in lipid consumption.

Physical activity prompts the movement of the fatty acid transporter (FAT/CD36) from its internal storage sites to the mitochondrial membrane in muscle cells (110, 111), where it engages with acyl-CoA synthetases that regulate the presence of fatty acyl-CoA for CPT1 (112), This indicates that FAT/CD36 could play a role in affecting how fatty acids are oxidized in mitochondria when engaging in physical exercise. The decomposition of fat could also be restricted by elevated malonyl-CoA, which is generated through a reaction facilitated by acetyl-CoA carboxylase (ACC). In lab environments, the compound functions as a regulator by altering the activity of CPT1 through an allosteric process (113). There are two variants of ACC: ACC1, which is also referred to as ACCα, and ACC2, which is also known by the name ACCβ. These variants can be found in various tissues, especially in skeletal muscle, where they are affected by hormonal signals and the availability of nutrients (114, 115). During intense physical activity, the activity of ACCβ in skeletal muscles diminishes, leading to decreased levels of malonyl-CoA, which in turn boosts the oxidation of fatty acids (116, 117), occasionally coinciding with a rise in the activity of 5’ AMP-activated protein kinase (AMPK) (118). On the other hand, deactivating ACC lowers malonyl-CoA concentrations, subsequently lessening the suppression of CPT1. This supports improved fatty acid breakdown as the body shifts from a state of rest to engaging in physical exercise (119). Thus, altering the activities of ACC and CPT-1 can affect the balance between how much intramuscular fatty acids are burned for energy and how much are stored as triacylglycerol.

In a 70-minute session on an ergometer cycle, which includes cycling for 10 minutes at 40% intensity before continuing for 60 minutes at 65% of VO2max, there is an increase in the breakdown of fatty acids. Nevertheless, the level of malonyl-CoA in skeletal muscle does not vary (120). As an alternative approach, engaging in one minute of cycling on an ergometer at 35% of VO2max results in a reduction of malonyl-CoA levels. However, these levels revert to their original state within 10 minutes. It’s interesting to note that while exercising at 65% of VO2max, malonyl-CoA levels do not fluctuate. Therefore, lowering malonyl-CoA levels is unnecessary to improve the absorption and processing of free fatty acids (FFA) during physical activity at 35% and 65% of VO2max. Additionally, because malonyl-CoA levels stay constant during exercises conducted at 90% VO2max, they are not involved in decreasing the rate of fat oxidation at this intensity (121). Furthermore, since malonyl-CoA levels remain stable during exercises performed at 90% VO2max, they do not play a role in reducing the rate of fat oxidation at this level of intensity. This decrease is linked to AMPK adding a phosphate group to ACCβ at the Ser221 location (122). Peroxisome proliferator-activated receptors (PPARs) are a group of nuclear transcription factors that require ligands to function and play a crucial role in maintaining metabolic balance. A 12-week endurance training program was evaluated by researchers studying its effects on six lean women. They analyzed alterations in overall body fat decomposition, plasma fatty acid oxidation, and PPARα levels in their skeletal muscles before and after the training period. Participating in training results in a 25% rise in the total decomposition of fatty acids during a 90-minute cycling session performed at 50% of the peak oxygen uptake measured before training. Furthermore, it results in almost doubling the levels of PPARα in muscle tissue. Consequently, this boosts the associated proteins involved in fatty acid decomposition, namely medium-chain acyl-CoA dehydrogenase (MCAD) and very long-chain acyl-CoA dehydrogenase (VLCAD) (75). Studies involving laboratory rodents have shown that PPARβ is crucial for sustaining slow-twitch oxidative fibers in skeletal muscles. A lack of it in these muscles could result in obesity and diabetes (123). Physical activity boosts the expression of PPARδ in skeletal muscles in both humans and rodents (124–126) PPARδ decreases the expression of genes involved in glycolysis within muscle tissue, resulting in reduced glucose consumption in mice (127).

**Figure 4:**
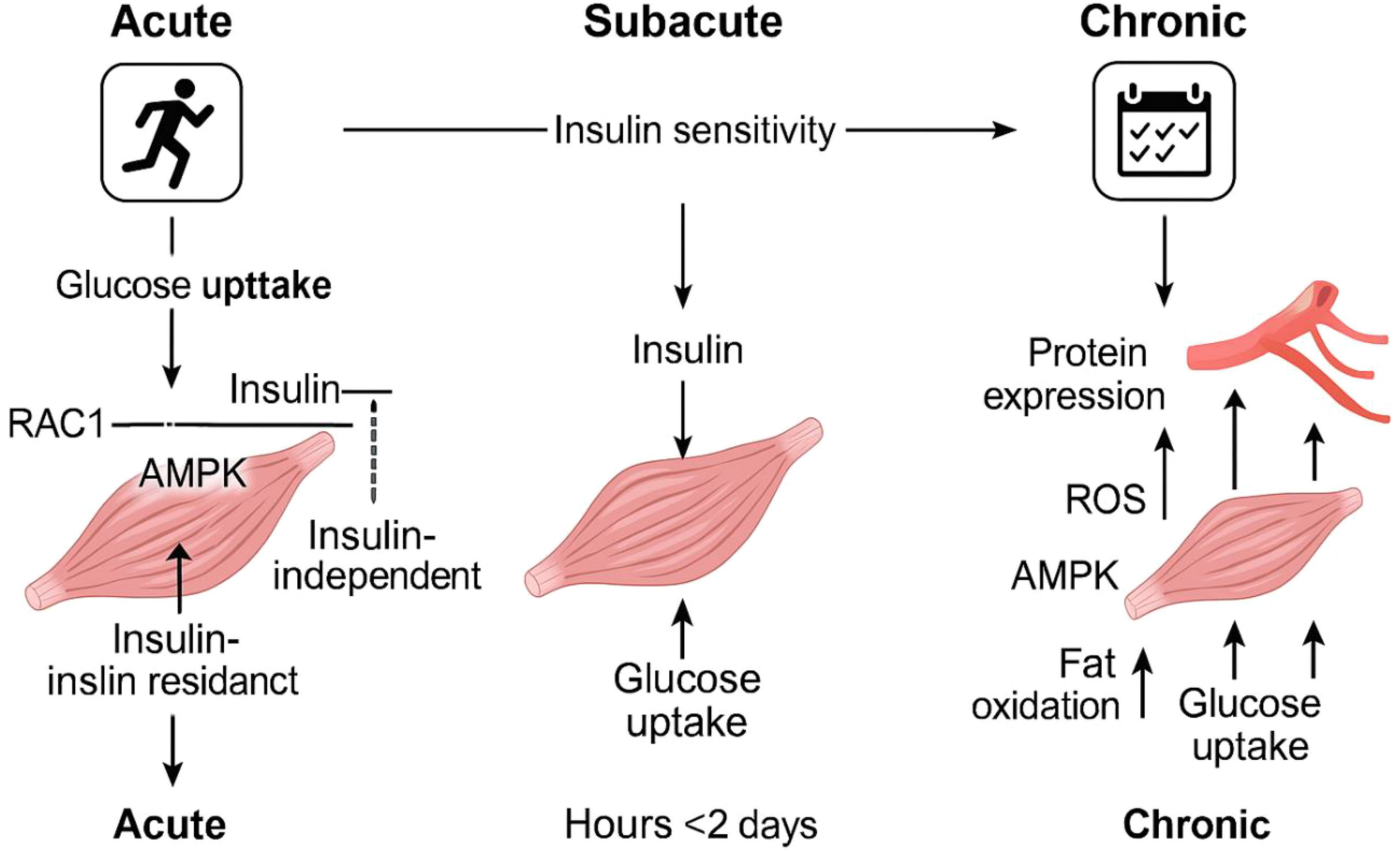
Diagram depicting the time-dependent metabolic advantages of physical activity and the associated molecular processes. One workout session temporarily boosts muscle glucose uptake without depending on insulin, and this effect is still observed in people who have insulin resistance. After engaging in physical activity, the body’s sensitivity to insulin, as evidenced by insulin-driven glucose uptake, temporarily enhances for as long as two days. Engaging in regular exercise results in long-term changes, such as heightened production of proteins involved in managing fat and glucose, and enhanced capillary growth. These changes improve the muscles’ capacity to utilize fat and glucose while increasing insulin sensitivity. The various abbreviations refer to adenosine monophosphate-activated protein kinase (AMPK), fatty acids (FA), Ras-related C3 botulinum toxin substrate 1 (RAC1), reactive oxygen species (ROS), and TBC1 Domain Family Member 1 (TBC1D1).

Research indicates that engaging in vigorous physical activity boosts the levels of proteins that transport fatty acids in skeletal muscles (128). Talanian et al. (128) noticed a rise in FABPpm and FAT/CD36 levels in a group of ten women who were not trained. Following six weeks of training, there is a significant rise in FAT/CD36 levels, with a 10% increase observed in the entire muscle and a substantial 51% increase in the mitochondrial membrane. However, no notable change is detected in the sarcolemmal membrane. The FABPpm levels rose by 48% in the entire muscle and by 23% in the sarcolemmal membrane, while there was no change in the mitochondria. Furthermore, according to Bradley and colleagues (129) showed that prolonged cycling at 60% of maximum oxygen uptake leads to increased amounts of FAT/CD36 and FABPpm proteins in the plasma membrane of human skeletal muscle. AMPK might play a role in facilitating the movement of FAT/CD36 and FABPpm to the cell surface (129, 130). Jeppesen et al. (131) noticed the absence of the liver kinase B1 protein (LKB1), which plays a crucial role as the primary enzyme responsible for enabling the phosphorylation of AMPK (132), greatly reduces the breakdown of fatty acids in mice during physical exercise and in isolated muscle contractions outside the body, indicating that LKB1 plays a vital role in muscle fatty acid oxidation during activity, independent of AMPK’s effect (131). **Figure 4** also demonstrates the short-term metabolic advantages of exercise along with the associated molecular mechanisms (**Figure 4**).

## Modulation of lipid metabolism by exercise in cancer: current research and therapeutic potentials

Numerous and reliable studies indicate that boosting physical activity significantly lowers the risk of developing cancer. Physically fit women, considering the limitations of observational studies, might experience as much as a 21% decrease in the rate of cancer occurrence (133). Randomized controlled trials are beginning to appear, and a few have demonstrated encouraging outcomes (134). Besides shedding pounds by altering one’s diet, participating in physical exercise is evidently a crucial element in lowering the risk of death associated with cancer. The processes involved are akin to those that might be influencing how obesity plays a part in cancer development. Recent research suggests that inflammation plays a role, specifically highlighting alterations in IL15 receptor interactions within CD8+ T cells that invade tumors (135). The suppression of tumor growth triggered by exercise in patient-derived xenograft models has been linked to natural metabolic shifts within cells. These changes predominantly involve modifications in mitochondria that impact oxygen utilization and the tricarboxylic acid (TCA) cycle (136). These mechanisms also suggest that physical activity might enhance the effectiveness of immune therapies or medications aimed at cancer metabolism. Therefore, gaining deeper insight into the fundamental processes is crucial to improve the chances of leveraging exercise as a preventive or therapeutic measure. For example, the way in which changes in muscle and fat mass, resulting from physical activity, affect tumor growth is not well comprehended. A clinical and mechanistic issue that remains unresolved is how to determine the appropriate dosage of exercise and to what degree this should be personalized for individuals (137). In individuals with prostate cancer, a home-based exercise therapy lasting two years (138) or a 12-week endurance training program (139) The intervention reduces the amount of body fat (138, 139) and triglycerides in the blood plasma (138). In a study using a rodent cancer model with high blood lipid levels, aerobic exercise training reduced the raised concentrations of plasma triacylglycerol and LDL and boosted HDL levels (140). While it hasn’t been explored specifically in relation to cancer, enhancing the insulin sensitivity of adipose tissue through exercise might reduce the heightened rate of fat breakdown linked to cancer, potentially safeguarding against muscle and tissue loss associated with cancer cachexia. This is because inhibiting fat breakdown has been shown to prevent tissue loss in rodent studies (141, 142). Finally, in mice, the issues of glucose and insulin intolerance linked to cancer can be improved by blocking fatty acid oxidation and inhibiting lipolysis (143), investigating the rationale for utilizing physical activity to control hyperlipidemia in individuals with cancer. Clinical trials are essential for evaluating how effective it is in humans. Hojman and her team aimed to clarify how voluntary wheel running influences tumor development control under both regular and high-fat dietary conditions. The inclusion of running wheels resulted in a 50% decrease in tumor growth in B16F10 tumor-bearing mice on a regular diet, whereas mice on a high-fat diet saw a 75% decrease (p < 0.001). Conversely, mice that were given a diet rich in fats exhibited a 53% rise in tumor development, with these findings being statistically significant (p < 0.001). The tumor size growth was linked to blood levels of glucose, leptin, and ghrelin, while no connection was found with insulin levels in the blood. Participating in voluntary wheel running boosted the immune system’s ability to detect tumors, according to microarray and gene expression research. This research identified markers linked to macrophages, natural killer (NK) cells, and T cells. However, a fat-rich diet resulted in a decrease in the previously high levels of macrophages and natural killer cells. Furthermore, research discovered that engaging in wheel running stimulated ZBP1, a protein involved in regulating innate immunity, whereas a high-fat diet inhibited its activity. Moreover, ZBP1 was associated with the recognition of innate immune responses in B16F10 tumors. Our study did not find any indication that ZBP1 influences cell cycle disturbances or necrosis caused by exercise in tumors of mice engaged in running activities. In conclusion, our findings align with epidemiological research indicating that physical activity might reduce tumor growth, regardless of BMI. However, our results indicate that consuming a diet high in fats could potentially diminish the ability of exercise to boost the immune system’s tumor recognition capabilities (144). A comprehensive assessment and meta-analysis were conducted to examine the impact of combining aerobic exercises with resistance training (AET + RET) versus conventional therapy in individuals diagnosed with breast cancer. Integrating aerobic and resistance exercises improved cardiopulmonary performance, as shown by a rise in VO2peak (mean improvement = 2.93 mL/kg/min; 95% confidence interval: 0.38 to 5.49; P-value = .02) and an increase in VO2max (mean improvement = 6.98 mL/kg/min; 95% confidence interval: 2.04 to 15.92; P-value = .01). Additionally, this combination resulted in a reduction in triglyceride levels (mean difference = -57.95 mg/dL; 95% CI: -112.25 to -3.64; P = .04), suggesting a beneficial effect on blood lipid profiles. No notable differences were observed between the AET + RET group and the standard care group in terms of peak heart rate, peak respiratory exchange ratio, systolic and diastolic blood pressure, high-density lipoprotein cholesterol, and body mass index, with a significance threshold of P < 0.05. The group that received AET + RET showed no notable differences compared to the standard care group in terms of peak heart rate, peak respiratory exchange ratio, systolic and diastolic blood pressure, HDL cholesterol levels, and body mass index, as all P-values were under.05, indicating no statistical significance (145).

In a different study, mice were divided into three separate categories: the first group served as a control (C), the second included mice that participated in exercise training but stopped when tumor cells were introduced (ET), and the third group continued their exercise routine throughout the entire study (EC). In the EC group, the solid leukemia tumors were significantly smaller than those in the C and ET groups, with a notable statistical significance (P < 0.05). In the tiniest tumors, lipid peroxidation levels appeared to rise, but these levels decreased as the tumors grew larger. It seems consistent that small tumors exhibit elevated lipid peroxidation levels, as this rise could actually impede cellular proliferation. Furthermore, scientists found that smaller tumors showed increased levels of I-Kb and Ras proteins. This suggests variations in the redox signaling pathway and implies a connection between redox signaling and tumor growth progression growth (146). **Table 1** provides an overview of changes in lipid metabolism during physical activity in relation to cancer.

**Table 1:** Different aspects of lipid metabolisms in cancer and impact of exercise on it.

Aspect	Exercise Effects
Fatty Acid Oxidation (FAO)	Exercise enhances fatty acid oxidation in skeletal muscle, reducing lipid availability for tumor cells.
Fatty Acid Synthesis	Exercise reduces *de novo* fatty acid synthesis by inhibiting key enzymes like ACC and FASN.
Key Enzymes Involved	Exercise upregulates CPT1, FASN, and FAT/CD36, promoting lipid oxidation and reducing metabolic flexibility in tumors.
Exercise Effects on Immune Function	Exercise improves immune function by enhancing the activity of immune cells, such as macrophages and T cells, in the tumor microenvironment.
Impact on Tumor Metabolism	Exercise disrupts the metabolic reprogramming of tumors, reducing their reliance on lipids for energy and growth.
Exercise and Cancer Therapy Integration	Exercise can complement cancer treatments by improving metabolic health, reducing cancer-related fatigue, and enhancing the efficacy of conventional therapies.

Taken all together, Exercise is known to significantly alter lipid metabolism by promoting fatty acid oxidation (FAO) in skeletal muscle, thereby decreasing the availability of lipids in circulation that may be utilized by tumor cells. During moderate-intensity exercise, the rate of FA oxidation in skeletal muscle can increase up to 10 times compared to resting conditions, contributing to a decrease in circulating lipids that can be available for tumor cells. Fatty acids, released from adipose tissue through lipolysis, are transported to muscle tissue, where they undergo oxidation to generate ATP. In cancer, tumor cells often rely on altered lipid metabolic pathways to sustain rapid growth, utilizing both *de novo* lipogenesis and exogenous lipid uptake for survival. Exercise, by enhancing FA oxidation and reducing lipogenesis, could potentially disrupt the lipid supply available to tumors, slowing their growth and progression. Key enzymes involved in lipid metabolism, such as acetyl-CoA carboxylase (ACC), fatty acid synthase (FASN), and carnitine palmitoyltransferase 1 (CPT1), which play pivotal roles in FA synthesis and oxidation, are all modulated by exercise. Exercise induces a decrease in the activity of ACC, leading to a reduction in malonyl-CoA levels, an inhibitor of CPT1. This reduction in malonyl-CoA levels facilitates the transport of fatty acyl-CoA into mitochondria for FAO. Furthermore, exercise upregulates the expression of fatty acid transport proteins (FAT/CD36) in skeletal muscle, enhancing FA uptake and oxidation. This shift from glucose metabolism to lipid metabolism during exercise further decreases the availability of glucose for tumor cells and reduces their metabolic flexibility.

Additionally, exercise increases mitochondrial function in muscle cells, promoting the oxidation of fatty acids to support ATP production. Enhanced mitochondrial activity can also lead to improved cellular energy homeostasis, reducing the energy available for tumor growth. Furthermore, exercise-induced mitochondrial biogenesis might make cancer cells more susceptible to oxidative stress, potentially inducing cell death. Studies have shown that FA oxidation also supports the generation of NADPH, a critical molecule for maintaining redox balance in cells. This generation of NADPH via FAO could improve immune function and limit tumor progression by enhancing the ability of immune cells to respond to tumors. Exercise may emerge as a viable therapeutic strategy in cancer treatment, especially when combined with other treatments such as chemotherapy or immunotherapy. The modulation of lipid metabolism by exercise offers a promising way to alter the metabolic landscape of tumors, making them less reliant on lipid-rich microenvironments. By reducing lipid availability and altering lipid composition in tumor cell membranes, exercise may help make tumors more susceptible to therapies targeting lipid metabolism or oxidative stress. Furthermore, exercise has the potential to improve the overall quality of life for cancer patients by reducing cancer-related fatigue, improving immune function, and counteracting the metabolic consequences of cancer, such as cachexia and insulin resistance. As part of a comprehensive cancer treatment plan, exercise could help optimize patient outcomes and may serve as an adjunctive therapy to reduce recurrence rates and enhance survival.

**Figure 5:**
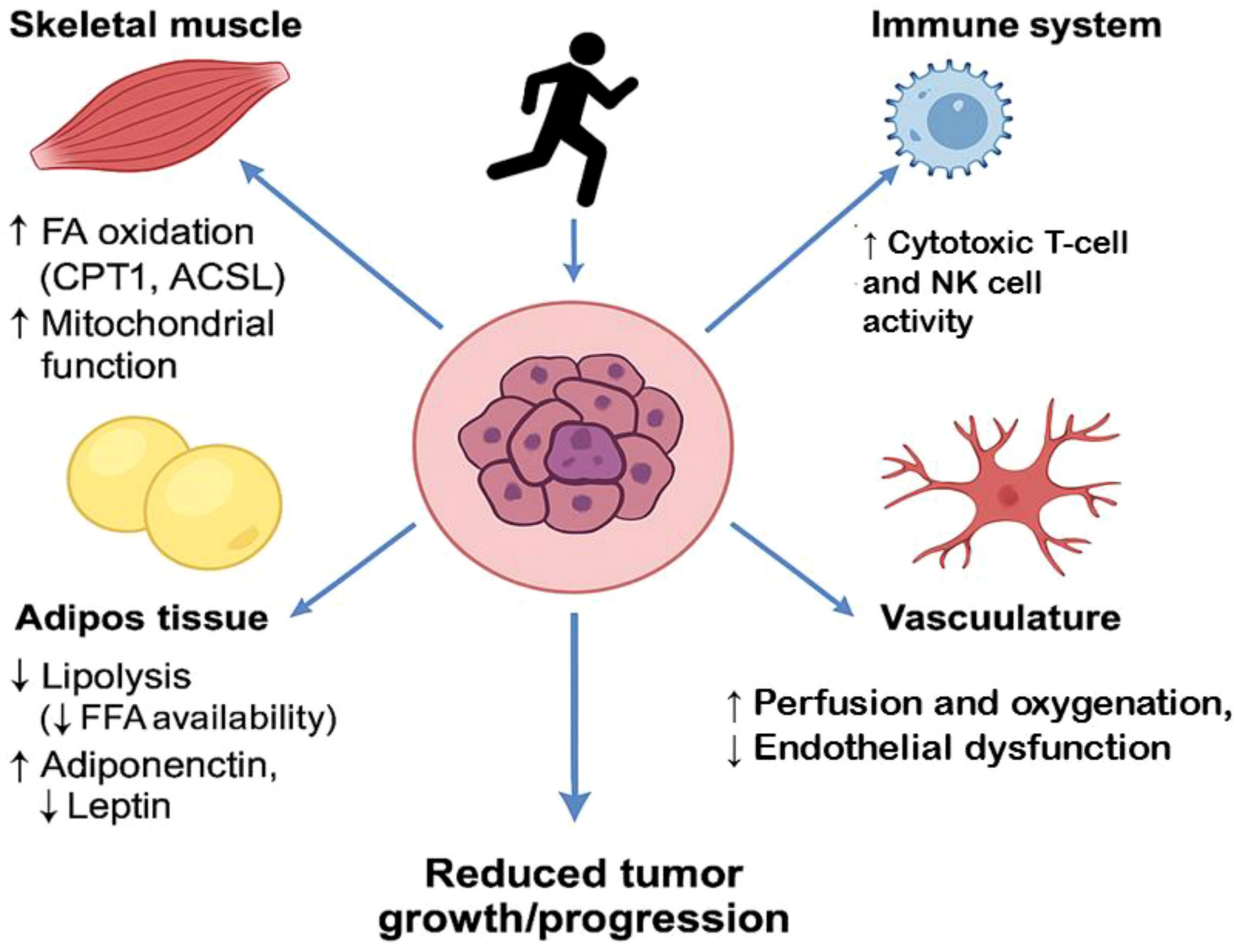
Systemic effects of exercise on tumor lipid metabolism. Skeletal Muscle: ↑ Fatty acid oxidation via CPT1 and ACSL1, ↑ Mitochondrial biogenesis and metabolic flexibility, contributes to reduced circulating lipids available to tumors. Adipose Tissue: ↓ Lipolysis, leading to decreased free fatty acid (FFA) spillover, ↑ Adiponectin, ↓ Leptin — improves metabolic and inflammatory tone, Reduces lipid supply and growth signals to the tumor. Immune System: ↑ Cytotoxic T-cell and NK cell activity via altered lipid handling, ↓ Immunosuppressive lipids (e.g., oxidized LDL, PGE2), Promotes anti-tumor immunity. Vasculature: ↑ Perfusion and oxygenation, improving nutrient flux and drug delivery, ↓ Endothelial dysfunction and aberrant angiogenesis, Impairs hypoxia-driven lipid synthesis in tumors, Tumor Microenvironment (Center): Receives reduced lipid support, Subjected to increased immune surveillance and less favorable metabolic conditions, Overall effect: ↓ Tumor growth and aggressiveness.

While the molecular mechanisms through which exercise influences lipid metabolism in cancer are becoming clearer, significant gaps remain in our understanding of the exact molecular processes and their full therapeutic implications. First, the precise dose-response relationship between exercise intensity, duration, and its effects on lipid metabolism in cancer cells needs to be further explored. Not all exercise modalities may be equally effective in modulating lipid metabolism or tumor progression, and current research has yet to determine the optimal exercise regimen for cancer patients based on tumor type, stage, and genetic makeup. Moreover, the heterogeneity of tumors and the complexity of the tumor microenvironment, including lipid availability, vascularization, and immune cell infiltration, complicate the understanding of how exercise impacts lipid metabolism in individual patients. Tumor cells can adapt to the loss of exogenous lipids through increased *de novo* lipogenesis, and it remains unclear how exercise can counteract this adaptive process in the context of various tumor types. To better understand the interplay between exercise and lipid metabolism in cancer, future research should focus on identifying specific biomarkers that can predict how exercise modulates lipid pathways in different cancer types. This research could also involve the use of advanced imaging techniques to assess lipid distribution and metabolism in tumors during exercise interventions. Investigating how exercise affects lipid metabolism in the tumor microenvironment, specifically within areas of poor vascularization or hypoxia, could provide valuable insights into how exercise may help overcome metabolic barriers in tumors. Additionally, more intervention studies are needed to establish the cause-effect relationship between exercise, lipid metabolism, and tumor suppression. These studies should examine the impact of various exercise regimens, both aerobic and resistance training, on lipid profiles, fatty acid oxidation, and tumor progression. Research should also explore the potential of combining exercise with drugs that target lipid metabolism, such as inhibitors of FASN, ACC, or CD36, to create synergistic effects that might further enhance cancer treatment. **Figure 5** shows a systemic effects of exercise on tumor lipid metabolism.

Endocrine-related tumors, such as breast and prostate cancers, exhibit complex interactions within their tumor microenvironment (TME), where lipid metabolism plays a pivotal role (147). Tumor cells often produce lipid mediators like prostaglandin E2 (PGE2) and oxidized low-density lipoprotein (oxLDL), which can suppress immune responses by impairing CD8⁺ T cell activation and promoting M2 macrophage polarization (148).​

Endothelial cells within the TME are also affected by lipid accumulation and hypoxia, leading to dysfunction characterized by increased vascular endothelial growth factor (VEGF) expression and enhanced angiogenesis. This aberrant vascular remodeling facilitates tumor progression and creates barriers to effective immune cell infiltration (148).​

Exercise has been shown to modulate these processes favorably. Regular physical activity enhances fatty acid oxidation in skeletal muscle, reducing circulating lipid levels that tumors might exploit (149, 150). Moreover, exercise mobilizes natural killer (NK) cells, bolstering innate immune surveillance. Additionally, exercise-induced improvements in vascular function can normalize tumor vasculature, potentially enhancing immune cell access to tumor sites (149, 150).​ These insights underscore the potential of integrating exercise into therapeutic strategies aimed at disrupting the lipid-mediated immunosuppressive and pro-angiogenic milieu of endocrine-related tumors.

To facilitate clinical translation, the identification of surrogate biochemical markers is essential for monitoring the impact of exercise on lipid metabolism and tumor progression. Circulating levels of oxidized low-density lipoprotein (oxLDL), prostaglandin E2 (PGE2), and leptin are emerging as markers of lipid-mediated inflammation and immunosuppression in the tumor microenvironment (150). Likewise, vascular endothelial growth factor (VEGF) and soluble intercellular adhesion molecule-1 (sICAM-1) may reflect endothelial dysfunction and pro-angiogenic activity, providing insight into angioinvasion and tumor aggressiveness (151, 152). Markers of oxidative stress such as 8-isoprostane, malondialdehyde (MDA), or glutathione redox ratios (GSH/GSSG) may also indicate the degree of metabolic stress within the tumor or systemic circulation (153, 154). Integrating these biomarkers with exercise interventions could help stratify patients based on metabolic risk and tumor phenotype, allowing for more personalized therapeutic approaches (155). Additionally, lipidomic profiling and functional imaging (e.g., FDG-PET, hyperpolarized carbon 13 MRI) could provide noninvasive tools to assess dynamic metabolic shifts during exercise-based therapy (39, 156).

## Conclusions

Exercise has emerged as a promising modulator of lipid metabolism in cancer, offering both therapeutic and preventive benefits. By enhancing fatty acid oxidation (FAO) and promoting mitochondrial function, exercise can limit the availability of lipids for tumor cells, disrupt metabolic reprogramming, and potentially slow tumor growth. Key enzymes and transporters involved in lipid metabolism, such as CPT1, FASN, and fatty acid transport proteins, are influenced by exercise, resulting in a shift from glucose metabolism to lipid oxidation, which may reduce the metabolic flexibility of cancer cells. Moreover, exercise enhances immune function and improves energy homeostasis, further supporting its role in cancer therapy. However, significant research gaps remain in understanding the precise molecular mechanisms and optimal exercise protocols tailored to different cancer types. Future studies should aim to establish the cause-effect relationship between exercise and tumor progression, investigate the synergistic effects of exercise and conventional therapies, and explore how exercise can be integrated into personalized cancer care. Despite these limitations, exercise holds substantial therapeutic potential and, when combined with traditional treatments, could significantly improve cancer management, quality of life, and patient outcomes. Recent evidence suggests that certain tumor types—particularly endocrine-related cancers such as breast and prostate—may be more responsive to exercise-based interventions due to their reliance on lipid metabolism. These tumors often exhibit elevated expression of lipid-related enzymes (e.g., FASN, ACC), transporters (CD36), and regulators (SREBP1), all of which support tumor growth, metastasis, and immune evasion. Patients with tumors exhibiting a high lipogenic index or vascular invasion may therefore derive greater benefit from exercise-driven metabolic modulation. Additionally, individuals with metabolic syndrome or obesity, conditions associated with elevated systemic lipid availability, may represent a particularly responsive subgroup. Future strategies may include biomarker-based profiling (e.g., lipidomics, imaging, gene expression) to tailor exercise prescriptions to the tumor’s metabolic phenotype, potentially enhancing therapeutic precision. To enhance clinical applicability, future research should prioritize integrative models that combine structured exercise interventions with multi-omic profiling and metabolic imaging. Targeted and untargeted lipidomics using LC-MS/MS can quantify circulating lipid species (e.g., ceramides, sphingolipids, acylcarnitines) pre- and post-exercise. These markers may act as surrogate indicators of tumor lipid uptake or systemic metabolic shifts. Tools like FDG-PET, 13C-pyruvate hyperpolarized MRI, or MR spectroscopy can capture exercise-induced changes in tumor metabolism. These methods may help visualize the switch from glycolysis to fatty acid oxidation *in vivo*. We propose a biomarker-embedded RCT framework: a)Intervention: aerobic and/or resistance training protocols (8–12 weeks), b) Participants: cancer patients (e.g., breast, colon, prostate), c) Endpoints: circulating lipids, tumor metabolic flux (via imaging), progression-free survival, quality of life, d) Exploratory arms: integration of muscle/adipose biopsies and tumor lipid signatures to examine localized responses.
